# Attributing changes in food insecurity to a changing climate

**DOI:** 10.1038/s41598-022-08696-x

**Published:** 2022-03-18

**Authors:** Shouro Dasgupta, Elizabeth J. Z. Robinson

**Affiliations:** 1 Centro Euro-Mediterraneo sui Cambiamenti Climatici (CMCC), Venice, Italy; 2 Università Ca’ Foscari Venezia, Venice, Italy; 3 Grantham Research Institute on Climate Change and the Environment, London School of Economics and Political Science (LSE), London, UK

**Keywords:** Climate-change impacts, Environmental economics

## Abstract

It is generally accepted that climate change is having a negative impact on food security. However, most of the literature variously focuses on the complex and many mechanisms linking climate stressors; the links with food production or productivity rather than food security; and future rather than current effects. In contrast, we investigate the extent to which current changes in food insecurity can be plausibly attributed to climate change. We combine food insecurity data for 83 countries from the FAO food insecurity experience scale (FIES) with reanalysed climate data from ERA5-Land, and use a panel data regression with time-varying coefficients. This framework allows us to estimate whether the relationship between food insecurity and temperature anomaly is changing over time. We also control for Human Development Index, and drought measured by six-month Standardized Precipitation Index. Our empirical findings suggest that for every 1 $^{\circ }\hbox {C}$ of temperature anomaly, severe global food insecurity has increased by 1.4% (95% CI 1.3–1.47) in 2014 but by 1.64% (95% CI 1.6–1.65) in 2019. This impact is higher in the case of moderate to severe food insecurity, with a 1 $^{\circ }\hbox {C}$ increase in temperature anomaly resulting in a 1.58% (95% CI 1.48–1.68) increase in 2014 but a 2.14% (95% CI 2.08–2.20) increase in 2019. Thus, the results show that the temperature anomaly has not only increased the probability of food insecurity, but the magnitude of this impact has increased over time. Our counterfactual analysis suggests that climate change has been responsible for reversing some of the improvements in food security that would otherwise have been realised, with the highest impact in Africa. Our analysis both provides more evidence of the costs of climate change, and as such the benefits of mitigation, and also highlights the importance of targeted and efficient policies to reduce food insecurity. These policies are likely to need to take into account local contexts, and might include efforts to increase crop yields, targeted safety nets, and behavioural programs to promote household resilience.

## Introduction

Food security, defined as existing when “all people, at all times, have physical and economic access to sufficient, safe and nutritious food that meets their dietary needs and food preferences for an active and healthy life”, depends on both food availability and food affordability^1^. Improvements in food insecurity at the global scale have long been closely linked to poverty reduction, as reflected in the World Bank’s poverty reduction strategy^2^. Yet recently at the global level a decoupling can be observed as the number of people in poverty continues on a downwards trend, while the proportion and absolute number of people experiencing food insecurity has started to increase over time^3,4^. Several explanations for this increase have been posited, including economic slowdowns; conflict; extreme weather events and climate variability; and, most recently, the COVID-19 pandemic^4–6^.

The mechanisms through which food security can be affected by climate change are many and often complex, and include the stability of and access to food supplies; impacts on prices, markets, and infrastructure across the the food chain; reduced incomes; and increases in the incidence of infectious and diarrhoeal diseases^7–9^. Yet while the potential links between food security and climate change have long been addressed in the academic literature^10,11^, a closer look reveals three important features. First, most articles purporting to identify links between climate change and food security focus on the narrower relationship between climate shocks and climate variability on agricultural output and food production^12–15^. For example, a reduction in consumable food calories has been attributed to changes in temperature and precipitation^16^. Other more complex mechanisms linking climate and food production are also being identified: for example, locust outbreaks, that can be devastating for crop production, have been found to be linked to long-term droughts, warm winters, and high spring and summer precipitation^17^. Links between pollinators and food security have also been identified^18^. Second, much of the literature is qualitative, focusing on pathways between climate change, food production, and food security. Third, most quantitative papers focus on future, rather than current impacts. For example, many papers use crop models, computable general equilibrium (CGE) models, and/or integrated assessment models (IAM) combined with with general circulation models (GCMs) to project the impact of future climate change on the population at risk of hunger^19–24^. However, there is a gap in the literature with respect to the identification of a plausible causal relationship between climatic stressors and food security indicators.

We present a novel and rigorous approach to determining the impact of climate change, as manifested in heat stress, defined as the temperature anomaly relative to a historic baseline, on food security. To do this, we had to overcome a number of substantive methodological challenges with regards to both data analysis and data. First, the relationship between climate and food security evolves and changes over time, and so a time constant regression framework, as is commonly used in the existing literature on climate and socioeconomic outcomes, is insufficient. We therefore use a time-varying regression, which can be tricky to operationalise and computationally intensive. Second, until recently, standardised data on food insecurity for a sufficient number of countries has been hard to access. In this paper, we sourced and merged data from the Food and Agriculture Organization (FAO) for 83 countries. Third, we had to define an appropriate measure of climate change. We chose to focus on temperature anomaly, defined as the annual deviation from a long-term rolling mean. Based on this approach, and controlling for other key factors that have been demonstrated to affect food insecurity, including extreme events, specifically droughts; and “development”, as proxied by sub-national HDI (the UNDP Human Development Index, disaggregated to the sub-national level); we are able to quantify the extent to which food security has already been negatively affected by climate change.

In this paper we make a step change contribution to the literature, providing for the first time a comprehensive quantitative assessment of the extent to which changes in food insecurity, an important driver of health, can be attributed to climate change. This is important for several reasons. First, our research provides additional evidence as to the broad health benefits of climate change mitigation, and as such increased support for global efforts to reduce carbon emissions. Climate change is increasingly being described as a health emergency^25,26^, and our paper demonstrates clearly that food security, including access to healthy and nutritious food, is harmed by increasing temperatures and increasing drought, and thus conversely, climate change mitigation will have a positive impact on food security. Second, a lack of empirical data has been highlighted as a key constraint, particularly for vulnerable countries, when it comes to designing policies and practices to address loss and damage, a focal area highlighted at the Glasgow COP26^27^. In this paper we are able to quantify for the first time the extent to which regions have differentially experienced loss and damage with respect to food security. Third, our research is important for discussions of climate justice. We find that African countries, which are least responsible for emissions, are experiencing the most negative impacts of climate change on food security. Fourth, the evidence generated in this paper can be used by policy makers to identify climate-food insecurity hotspots, to enable the design of more effective tailored policies that take into local contexts.

In the next section, we detail our methodological approach, in the context of the current attribution literature. We then present our empirical findings, highlighting the annual temperature anomaly and time-varying regression results, and analysis of the counterfactual of a no climate change scenario. In our final Section (4) we conclude, discussing the implications of our findings for future research and for practical food policy making.

## Methods and data

Attributing loss and damage to climate change, that is, being able to state the extent to which human induced climate change increases the probability of an event or outcome, is a relatively new and evolving literature. Allen^28^ was one of the first articles to explore the extent to which it is possible to attribute outcomes to climate change through its impact on weather. Since then, the number of attribution studies that quantify links between climate change and health has grown, driven in the main by climatologists, who use established formal detection and attribution methods to determine the extent to which climate change is affecting health^29^. These “D and A” methods are based on statistical and process-based approaches to determining a causal link between a hazard linked to climate, and a negative health outcome. For example^30^, focus on the links between heat extremes and mortality. They take temperature data for Stockholm, Sweden, defining extreme heat and cold event thresholds, and calculate the number of such extreme events in 1900-1929 and 1980-2009. Then, combining the long-term temperature data set with recent health data, the authors are able to attribute recent deaths from extremes of temperature to observed climate change, controlling for various confounders such as age and urbanisation. More recently, Vicedo and colleagues^31^ use mortality and weather data from 732 locations in 43 countries during 1991–2018 to attribute 37% of warm-season heat-related deaths to anthropogenic climate change. Mitchell and colleagues^32^ focus on the heatwave of 2003 and estimate that anthropogenic climate change increased the risk of heat-related mortality in Central Paris by 70% and by 20% in London.

Complementing this literature, but taking an approach grounded in applied econometrics, we combine newly available panel data on food insecurity, collected on a regular basis by FAO in collaboration with Gallop World Poll in a large number of countries at the individual level, with temperature anomalies data. Controlling for confounding factors, we are able to identify a plausible causal link between food insecurity and our changing climate.

### Empirical framework

To track the impact of climate change and inequality on incidence of food insecurity, we use a panel data regression with coefficients that vary over time. To operationalise the concept of climate change, we focus on temperature anomaly, defined as the annual temperature difference, in $^{\circ }\,\hbox {C}$, from a mean temperature of a 30-year period between 1981-2010. We consider two dependent variables: first, the probability of moderate to severe food insecurity; and second, the probability of severe food insecurity. We examine the impact of temperature anomaly on food insecurity, controlling for sub-national HDI and drought. We use six-month Standardized Precipitation Index (SPI) as a measure of drought, this is a common indicator of agricultural drought and as such appropriate for our analysis. To account for unobserved heterogeneity, our specification also includes both location and time (year) fixed-effects.

Most of the empirical literature focuses on constant-parameter coefficients that do not change over time. A standard fixed-effects (FE) specification can be written as:

1 $$\begin{aligned} y_{it} = \alpha _{i} + x^{\prime }_{it}\beta + \mu _{it} \end{aligned}$$

where $\beta$ is a time-constant coefficient that measures the marginal impact above cross-sectional units’ long-run average rate. A fixed-effects specification allows the individual and/or time specific effects to be correlated with explanatory variables. An assumption with such panel FE specifications is that the effects of observed explanatory variables, $\underline{x}$, are identical across cross-sectional units (*i*), and over time (*t*). However, this assumption of a time-constant effect of temperature anomaly may be too restrictive if the impact of climatic stressors on socioeconomic outcomes evolves over time, implying that more flexible approaches may therefore be needed. In such cases, regression specifications allowing for a time-varying association between the dependent variable and the covariates of interest are most likely more appropriate.

We posit that in the case of temperature anomaly and food insecurity, $\beta$ is likely to vary over time. That is, the temperature anomaly will have a differential impact on food insecurity in different years. We therefore estimate a plausible causal relationship between temperature anomaly and food insecurity using panel data models with coefficients that vary over time. More generally, time-varying specifications are useful to characterise non-constant relationships between predictors and responses in regression models^33^. These specifications allow estimation of coefficients that are common to all cross-sectional units and time; parameters that vary over cross-sectional units; and coefficients that change over time. Following^34^, the general form of a regression with a time-varying parameter can be written as follows:

2 $$\begin{aligned} y_{t} = \beta ^{\prime }_{t} x_{t} + \mu _{t} \end{aligned}$$

Here we are relaxing the assumption of a constant relationship across time between a set of control variables and a dependent variable. In effect, this allows us to estimate the extent to which, if at all, the relationship between food insecurity and temperature variability has evolved over time, by incorporating the dynamic pattern of this relationship. These specifications are computationally intensive and non-convergence issues are rather common. An econometric specification with time-varying coefficients and fixed-effects can be written as:

3 $$\begin{aligned} y_{it} = \sum _{k = 1}^{K}(\bar{\beta _{k}} + \alpha _{ki} + \lambda _{kt})x_{kit} + \mu _{it} \end{aligned}$$

where $y_{it}$ (the response variable) is the incidence of moderate to severe and severe food insecurity as measured by FIES; $\beta _{k}$ are coefficients that are constant over time and space; $\alpha _{ki}$ are coefficients that vary over cross-sectional units; $\lambda _{kt}$ are coefficients that vary over time; $x_{kit}$ are explanatory variable(s) (in our case temperature anomaly); while $\mu _{it}$ is the error term. Since the coefficient $\beta$ depends on time *t*, the modeling bias and the *curse of dimensionality* can be reduced to some extent^35,36^. In our case, this is interesting as we are able to study the extent to which the temperature anomaly affects food insecurity over time. Our regression specification can be written as follows:

4 $$\begin{aligned} FIES_{it} = \beta _{1}(\tau _{t})\varvec{V}_{it} + \gamma ^{\prime }(\tau _{t})\varvec{X_{it}} + \alpha _{i} + \mu _{it} \end{aligned}$$

Equation (4) is a panel data regression model with time-varying coefficients and both location and time fixed-effects, where $FIES_{it}$ is the probability of moderate to severe food insecurity, or probability of severe food insecurity; $\varvec{V}_{it}$ is the temperature anomaly; and $\varvec{X_{it}}$ is a vector of relevant variables affecting food insecurity including sub-national HDI and extreme events (droughts); while $\mu _{it}$ is a random error term. All variables are recorded for different locations with index *i* $=$ 1, ... , N and over a number of years *t* $=$ 1, ... , T. The time-varying coefficients allow us to examine whether the relationship between temperature anomaly and food insecurity has evolved over time.

### Data

We use prevalence of food insecurity data, based on the Food Insecurity Experience Scale (FIES)^37,38^, which provides internationally-comparable estimates of the proportion of the population facing difficulties in accessing food. The FIES-based indicators are compiled using the FIES survey module, containing eight questions, which are then used to compute the probabilities of moderate or severe food insecurity and severe food insecurity. FAO collects nationally representative samples of the adult population, once every year beginning in 2014, to develop methods to estimate cross-country comparable prevalence rates of moderate and severe food insecurity. FAO estimates a Rasch model-based scale for each country and data are assessed for consistency to ensure cross-country comparability. The following questions are asked in a FIES module to compute the probabilities of food insecurity.

Question: During the last 12 months, was there a time when, because of lack of money or other resources:

1. You were worried you would not have enough food to eat?
2. You were unable to eat healthy and nutritious food?
3. You ate only a few kinds of foods?
4. You had to skip a meal?
5. You ate less than you thought you should?
6. Your household ran out of food?
7. You were hungry but did not eat?
8. You went without eating for a whole day?

The responses to these questions are classified into: (1) moderate to severe food insecurity, which is associated with reduced quality and/or quantity of food consumption including eating fewer meals (question 4), eating smaller portions (question 5), and running out food (question 6); and (2) severe food insecurity, which is associated with a high probability of reduced food intake such as going hungry without eating (question 7), and not eating for an entire day (question 8). The raw data consists of 411,403 individual data points which are aggregated using the survey weights provided in the datasets. Naturally all household survey data may have biases, due to data collection collection relying on individual recall, and FIES is no exception to this.

For climate data, we use reanalysed data from ERA5-Land, the fifth generation European Centre for Medium-Range Weather Forecasts (ECMWF) atmospheric reanalysis of the global climate. Reanalysed climate data combines global climate models (numerical representation of the recent climate) with observational and satellite observations. Reanalysed data has the advantage of producing long time series and spanning the entire planet^39^. Data from ERA5-Land is available at a spatial resolution of $0.1^{\circ } \times 0.1^{\circ }$ and hourly temporal-resolution^40^. We extracted the climatic data for each region using georeferences before computing the mean annual temperature, 30-year (1981-2010) mean temperature, and finally the anomaly as a difference between the annual mean and the 30-year mean temperature for each region. We aggregate the number of times the six-month SPI is below the threshold of -1.5 in a given month to compute our drought indicator. Because we aggregate data to the annual level, temporal heterogeneities cannot be controlled for.

We also use the sub-national Human Development Index (SHDI) database^41^, which contains data from 163 countries, aggregating education, health, and standard of living dimensions. The SHDI, which comprises economic and social indicators, also allows us to incorporate within-country variation and inequality in wellbeing and its associated impact on food insecurity.

### Data statement

All methods were carried out in accordance with relevant guidelines and regulations. We use secondary data for our analysis. The surveys were conducted by the Gallup World Poll, who obtained informed consent from all the respondents. The datasets used were anonymised by removing all identifying information on households and individuals before being made available for research purposes.

## Findings

### Descriptive statistics

**Figure 1:**
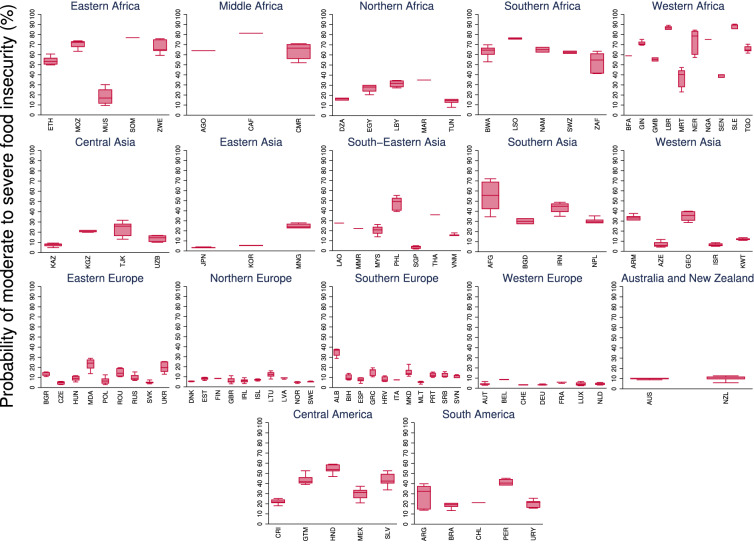
Probability of moderate to severe food insecurity (%) across regions. The global average during 2014–2019 was 22.7%.

**Figure 2:**
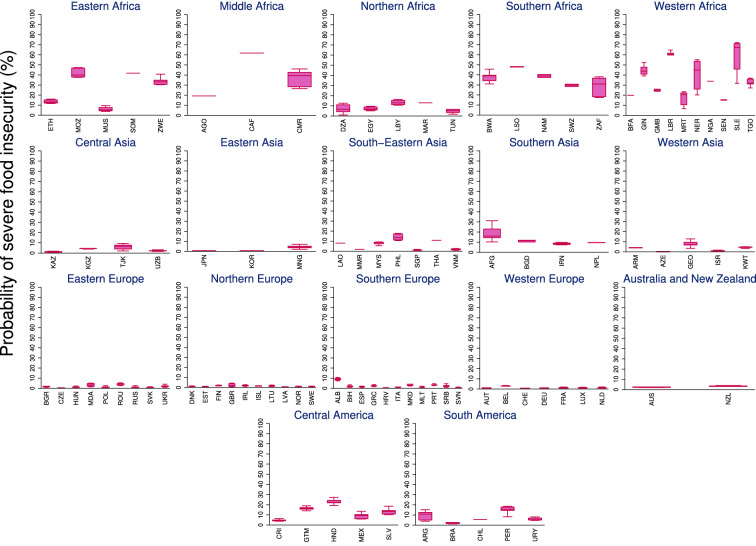
Probability of severe food insecurity (%) across regions. The global average during 2014–2019 was 7.9%.

We aggregated the food insecurity data into 17 sub-regions following the United Nations Geoscheme. The probability of moderate to severe food insecurity across the globe increased from 19.3% in 2014 to 30.7% in 2019 (Fig. 1). Nearly 11% of the population across 83 countries suffered from severe food insecurity in 2019, a significant increase from 6.2% in 2014 (Fig. 2). There are clear across and within-regional differences. For example, incidences of food insecurity are relatively higher in the Africa region, with Liberia, Guinea, and Mozambique reporting the highest levels of food insecurity. Honduras in the Americas, and Afghanistan and The Philippines in the Asia region, have also reported relatively high levels of food insecurity. While food insecurity is generally low in Europe, countries such as Albania, Moldova, and Ukraine have recently reported increasing levels of food insecurity. In terms of gendered impacts, 54% of the countries included in this analysis reported higher probability of food insecurity among women compared to men.

**Figure 3:**
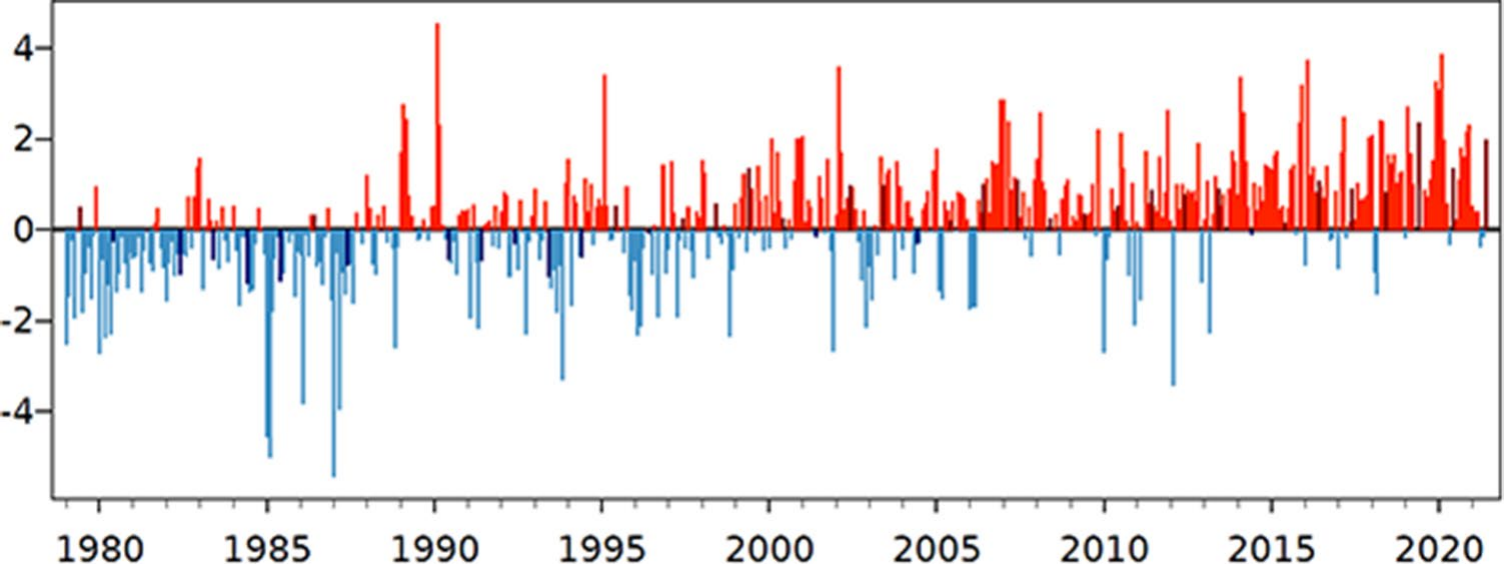
Monthly global temperature anomalies ($^{\circ }\hbox {C}$).

Globally, temperature anomaly has been increasing, and the countries in our sample have experienced a similar trend (Fig. 3). The mean temperature anomaly in our sample data is 0.56$^{\circ }\,\hbox {C}$. Our data also suggest that regions with the highest increases in temperature also tend to suffer from relatively high incidences of severe food insecurity.

### Empirical findings

**Figure 4:**
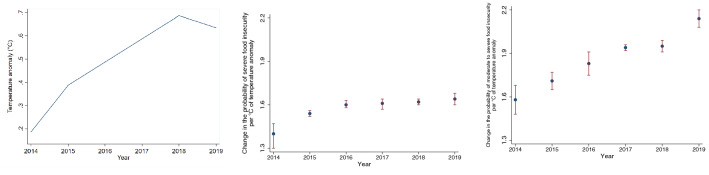
Annual temperature anomaly and time-varying regression results.

Our time-varying regression, that allows us to estimate the impact of temperature anomaly on food insecurity for the six consecutive years for which FIES data are available, suggests that for every 1$^{\circ }\,\hbox {C}$ of temperature anomaly, severe global food insecurity increased by 1.4% (95% CI: 1.3-1.47) in 2014 but by 1.64% (95% CI: 1.6-1.65) in 2019, suggesting an increasing trajectory (Table 1 and Fig. 4, second-panel). The impact of temperature anomaly on moderate to severe global food insecurity is higher, with the results suggesting that a 1$^{\circ }\,\hbox {C}$ increase in temperature anomaly increased moderate to severe food insecurity by 1.58% (95% CI: 1.48-1.68) in 2014 but had a significantly higher impact of 2.14% (95% CI: 2.08-2.20) in 2019 (Table 1 and Fig. 4, third-panel). We formally tested this difference using a multi-variate regression which provided statistical evidence that the impact of temperature anomaly on moderate to severe and severe food insecurity is heterogeneous. One of the advantages of using a time-varying coefficients regression is that we are able to identify the impact of temperature anomaly on food insecurity for every time-period in our dataset. Our approach reveals that temperature anomaly not only increases the probability of food insecurity but the magnitude of this impact is increasing over time. We tested this hypothesis using Wald tests, which suggest that the each of the coefficients in year $\textit{t}$ were grater than that in year ${t_{t-1}}$. These results are worrying, as they suggest that the temperature anomaly may continue to increase due to future climate change, likely further intensifying the stress on food security.

In our regression, the other variables that we control for, human development index, and drought, also show significant impacts on food insecurity (Table 1). Perhaps not surprisingly, regions with relatively higher HDI are associated with lower probability of food insecurity: each improvement of HDI of 0.1 (on a scale of 0 to 1) is associated with a 2.3% lower probability of severe food insecurity and 2.7% lower probability of moderate to severe food insecurity. Given that the increase in HDI for the median country over the 30-year sample period is only 0.11, our findings suggest that improvements in within-country wellbeing/reduction in inequality are likely to play an important role in reducing the incidence of food insecurity. Furthermore, our findings show that increasing frequency of droughts (SPI-6) increases the probability of both moderate to severe and severe food insecurity.

**Table 1:** Main regression results.

	Moderate to severe	Severe
SHDI	− 2.7	− 2.3
	(− 2.9, − 2.5)	(− 2.6, − 2.0)
Drought (SPI-6)	0.014	0.011
	(0.010, 0.018)	(0.008, 0.014)
Temperature anomaly$^{\mathrm{(t)}}$		
2014	1.58	1.40
	(1.48, 1.68)	(1.30, 1.47)
2015	1.71	1.54
	(1.65, 1.77)	(1.52, 1.56)
2016	1.83	1.60
	(1.75, 1.91)	(1.58, 1.63)
2017	1.94	1.61
	(1.92, 1.96)	(1.57, 1.64)
2018	1.95	1.62
	(1.91, 1.99)	(1.60, 1.64)
2019	2.14	1.64
	(2.08, 2.2)	(1.60, 1.68)
95% confidence intervals in parentheses		

### Robustness tests

Our results from the main specifications are consistent with a series of robustness tests. In the first robustness specification (Table 2), we extend our main specification with time-varying bins of monthly temperature anomalies. Using the 0.2–0.4$^{\circ }\,\hbox {C}$ as the reference bin, our results suggest that relatively low monthly temperature anomalies (< 0.2$^{\circ }\,\hbox {C}$) reduce incidences of food insecurity (for both indicators). However, the coefficients for this anomaly bin changes rather slowly over time. Compared to the reference bin, temperature anomalies in the higher bins result in an increase in incidences of food insecurity. The coefficients of all the higher temperature bins (additional months with relatively higher temperature anomalies) also increase over time, providing further evidence that the magnitude of increasing temperature anomaly on food insecurity has increased over time.

**Table 2:** Time-varying regressions with bins of monthly temperature anomalies (0.2–0.4$^{\circ }\,\hbox {C}$ is the reference bin).

	Moderate to severe	Severe
SHDI	− 2.631	− 2.147
	(− 2.848, − 2.414)	(− 2.292, − 2.002)
Drought (SPI-6)	0.014	0.008
	(0.012, 0.016)	(0.007, 0.009)
Temperature anomaly_(t)_		
2014: $< 0.2^{\circ }\,\hbox {C}$	− 0.008	− 0.005
	(− 0.007, − 0.011)	(− 0.004, − 0.006)
2015: $< 0.2^{\circ }\,\hbox {C}$	− 0.009	− 0.006
	(− 0.008, − 0.010)	(− 0.003, − 0.009)
2016: $< 0.2^{\circ }\,\hbox {C}$	− 0.009	− 0.005
	(− 0.007, − 0.011)	(− 0.003, − 0.007)
2017: $< 0.2^{\circ }\,\hbox {C}$	− 0.008	− 0.006
	(− 0.004, − 0.011)	(− 0.003, − 0.009)
2018: $< 0.2^{\circ }\,\hbox {C}$	− 0.010	− 0.005
	(− 0.008, − 0.012)	(− 0.004, − 0.006)
2019: $< 0.2^{\circ }\,\hbox {C}$	− 0.009	− 0.006
	(− 0.007, − 0.011)	(− 0.004, − 0.007)
2014: 0.4–0.6$^{\circ }\,\hbox {C}$	0.028	0.020
	(0.026, 0.030)	(0.018, 0.022)
2015: 0.4–0.6$^{\circ }\,\hbox {C}$	0.030	0.021
	(0.027, 0.033)	(0.017, 0.025)
2016: 0.4–0.6$^{\circ }\,\hbox {C}$	0.031	0.022
	(0.030, 0.032)	(0.019, 0.024)
2017: 0.4 –0.6$^{\circ }\,\hbox {C}$	0.033	0.024
	(0.031, 0.035)	(0.022, 0.026)
2018: 0.4 –0.6$^{\circ }\,\hbox {C}$	0.033	0.024
	(0.030, 0.036)	(0.021, 0.027)
2019: 0.4 –0.6$^{\circ }\,\hbox {C}$	0.035	0.026
	(0.033, 0.037)	(0.024, 0.028)
2014: 0.6–0.8$^{\circ }\,\hbox {C}$	0.039	0.030
	(0.037, 0.041)	(0.028, 0.032)
2015: 0.6–0.8$^{\circ }\,\hbox {C}$	0.040	0.031
	(0.039, 0.041)	(0.029, 0.033)
2016: 0.6–0.8$^{\circ }\,\hbox {C}$	0.041	0.033
	(0.039, 0.043)	(0.031, 0.034)
2017: 0.6–0.8$^{\circ }\,\hbox {C}$	0.043	0.035
	(0.040, 0.046)	(0.033, 0.037)
2018: 0.6 –0.8$^{\circ }\,\hbox {C}$	0.042	0.034
	(0.040, 0.044)	(0.031, 0.037)
2019: 0.6–0.8$^{\circ }\,\hbox {C}$	0.044	0.036
	(0.043, 0.045)	(0.033, 0.039)
2014: $> 0.8^{\circ }\,\hbox {C}$	0.047	0.039
	(0.044, 0.050)	(0.037, 0.041)
2015: $> 0.8^{\circ }\,\hbox {C}$	0.048	0.041
	(0.046, 0.050)	(0.038, 0.044)
2016: $> 0.8^{\circ }\,\hbox {C}$	0.050	0.042
	(0.047, 0.053)	(0.039, 0.045)
2017: $> 0.8^{\circ }\,\hbox {C}$	0.051	0.043
	(0.049, 0.053)	(0.040, 0.046)
2018: $> 0.8^{\circ }\,\hbox {C}$	0.050	0.043
	(0.047, 0.053)	(0.042, 0.044)
2019: $> 0.8^{\circ }\,\hbox {C}$	0.054	0.045
	(0.052, 0.056)	(0.043, 0.047)

We also run a binned regression using an OLS specification with fixed effects (Table 3). These results further show that, compared to the 0.2–0.4$^{\circ }\,\hbox {C}$ temperature anomaly bin, if there are more months with relatively higher temperature anomalies there is a greater incidence of food insecurity, while if there are more months with relatively lower temperature anomalies (<0.2$^{\circ }\,\hbox {C}$) compared to the reference bin results, the incidence of food insecurity is lower.

**Table 3:** Regressions with bins of monthly temperature anomalies (0.2–0.4$^{\circ }\,\hbox {C}$ is the reference bin).

	Moderate to severe	Severe
SHDI	− 2.642	− 2.327
	(− 2774, − 2.600)	(− 2.443, − 2.211)
Drought (SPI-6)	0.014	0.010
	(0.012, 0.016)	(0.008, 0.012)
$< 0.2^{\circ }\,\hbox {C}$	− 0.009	− 0.007
	(− 0.008, − 0.010)	(− 0.005, − 0.009)
0.4–0.6$^{\circ }\,\hbox {C}$	0.014	0.012
	(0.012, 0.016)	(0.011, 0.013)
0.6 –0.8$^{\circ }\,\hbox {C}$	0.020	0.016
	(0.018, 0.022)	(0.014, 0.018)
$> 0.8^{\circ }$	0.028	0.020
	(0.027, 0.030)	(0.018, 0.022)
95% confidence intervals in parentheses		

### Counterfactual analysis

We conduct a counterfactual analysis to explore the extent to which historical climate change may have negated potential improvements in food security. To do this we compute the cumulative impacts of temperature anomaly above the historical norms over the period 1981–2010. We use data from the Detection and Attribution Model Intercomparison Project (DAMIP)^42^ of the Coupled Model Intercomparison Project Phase 6 (CMIP6), merged with SSP2-RCP4.5 (considered a “middle of the road” scenario) runs of twelve GCMs from CMIP6. The counterfactual impact of climate change on food insecurity is derived by comparing the outputs from Equation 4 of region *i* over these two scenarios. We consider the effects of sub-national region-specific average annual temperature increases over the 2014–2019 period compared to the baseline scenario (1981–2010) under which temperature in each region increases according to its historical trend.

Our counterfactual analysis for moderate to severe food insecurity (Table 4; columns 2–3) shows that incidence of food insecurity would have been 47.65% in Africa (2.25 percentage-points lower) if the temperature followed the historical trajectory. The lowest change would have been in Europe, where food insecurity would have been 11.73% without climate change compared to 13.19% with climate change (1.46 percentage-points lower). For the case of severe food insecurity (Table 4; columns 4–5), the cumulative effects of climate change are smaller but still non-negligible. In Africa, severe food insecurity would have been 21.8% if the temperature followed the historical trajectory (0.88 percentage-points lower) while the lowest estimated change would have again been in Europe, 1.86% compared to 2.05% (0.19 percentage-points). These differences in impacts are driven by the differentiated impacts of temperature anomaly on the two indicators of food insecurity (Fig. 4).

**Table 4:** Counterfactual analysis: effects of climate change on food insecurity.

Counterfactual analysis: effects of climate change on food insecurity				
Region	Moderate to severe (historical)	Counterfactual scenario	Severe (historical)	Counterfactual scenario
Africa	49.89%	47.65%	22.68%	21.80%
Americas	32.72%	30.72%	10.67%	10.11%
Asia	33.49%	31.35%	9.69%	9.10%
Europe	13.19%	11.73%	2.05%	1.86%

## Discussion

The links between climate change and food security are complex and many, and are well documented in the literature. The Intergovernmental Panel on Climate Change states with “high confidence” that climate change is already affecting food insecurity across the globe^43^. Yet there are still insufficient attempts to quantify this relationship and explore the extent to which it is possible to attribute changes in food security to climate change. Rather, most of the literature addressing climate change impacts focuses on crop yields, production, and productivity, and future impacts of climate change, with much less attention given to the negative impact on food security that is already occurring^44^. Our paper makes three important and distinct contributions to these gaps in the literature.

First, we provide the first global and comprehensive quantitative assessment of the extent to which climate change is already having a measurable impact on food security, and our findings are sobering. We track the link between temperature anomaly, drought, and food insecurity, using a relatively new dataset collected by FAO since 2014, that focuses on people’s lived experiences of food insecurity, such as whether they had to skip meals, worried about not having enough to eat, or were not able to eat healthy and nutritious meals. We provide quantitative evidence that climate change, proxied by temperature anomaly and drought, is already having a negative impact on food security across the four regions of Africa, Americas, Asia, and Europe. Our findings are consistent with the literature that focuses on the links between climate change and food production^12–15^. Importantly, we additionally find that the impact of the temperature anomaly on food insecurity is increasing over time, as average temperatures increase. Though this pattern is particularly pronounced for moderate to severe food insecurity, a similar pattern can be found for severe food insecurity. Because we focus on food security rather than food production, our findings have particular relevance for economic development and poverty reduction more broadly.

While it does not come as a surprise that warming worsens food security in most countries (as has been shown to be the case for Ethiopia^11^), given the lack of quantitative evidence in the existing literature it is difficult to compare the effect size. However, by undertaking a counterfactual analysis, we are able to quantify the extent to which climate change is reversing the gains in food security that would otherwise have been realised, most likely through policies addressing poverty reduction and economic growth. We find that for all four regions, climate change appears to have had a non-trivial impact on food insecurity. Though the changes may appear relatively small, these changes have occurred over a short period of time, and the negative impacts on food security are likely to increase yet further as temperatures continue to rise. Our findings contribute to the growing focus on loss and damage, and the extent to which climate stressors are affecting Sustainable Development Goals, including food security^45^. Our findings may also provide a partial explanation as to why, despite falling global poverty rates, both the percentage and the absolute number of undernourished people have started to increase^4^.

Third, though food insecurity in each of the four regions appears to have increased due to climate change, these impacts are heterogeneous. In particular, our counterfactual analysis suggests that Africa, already the most food insecure region, has been hardest hit with regards to the impact of climate change on food insecurity. Our analysis suggests that, between 2014 and 2019, severe food insecurity is 0.88 percentage-points higher, and moderate to severe food security 2.24 percentage-points higher, due to climate change. These results do not surprise us, African countries have long been identified as being particularly vulnerable to the impacts of climate change on food security^46–48^.

Our paper makes clear that climate change is reversing efforts, particularly in lower-income countries, to reduce poverty and increase prosperity, providing yet more evidence for the urgent need for global reductions in carbon emissions. Yet the reality, that climate change is already worsening both moderate and severe food security, across all regions, also highlights the need for greater attention to adaptation, building resilience, and addressing loss and damage.

For policy makers, it is important to understand not just whether climate change is affecting food security, but how, so that policies can be targeted effectively. For example, in some regions the focus might be on smallholder agricultural productivity, including investments in soil quality; in others safety nets such as food or cash transfers; or improvements in the food supply chain and regional storage^11^. Such policies and approaches are likely to be location specific, informed by detailed country-specific studies, involving local researchers, policy makers, and civil society.

## Data Availability

The data used in this paper are publicly available at https://microdata.fao.org/index.php/catalog.
